# Distinctive clinical and imaging trajectories in SWEDD and Parkinson’s disease patients

**DOI:** 10.1016/j.nicl.2024.103592

**Published:** 2024-03-14

**Authors:** Cecilia Boccalini, Nicolas Nicastro, Daniela Perani, Valentina Garibotto

**Affiliations:** a Vita-Salute San Raffaele University, Milan, Italy; b IRCCS San Raffaele Scientific Institute, Milan, Italy; c Laboratory of Neuroimaging and Innovative Molecular Tracers (NIMTlab), Geneva University Neurocenter and Faculty of Medicine, University of Geneva, Geneva, Switzerland; d Division of Neurorehabilitation, Department of Clinical Neurosciences, Geneva University Hospitals, Geneva, Switzerland; e Nuclear Medicine Unit, San Raffaele Hospital, Milan, Italy; f Division of Nuclear Medicine and Molecular Imaging, Geneva University Hospitals, Geneva, Switzerland; g CIBM Center for Biomedical Imaging, Geneva, Switzerland

**Keywords:** ^123^I-FP-CIT SPECT, Neurotransmission, Scans without evidence of dopaminergic deficit (SWEDD), Parkinson's disease, Molecular connectivity

## Abstract

A proportion of patients clinically diagnosed with Parkinson’s disease (PD) can have a ^123^I-FP-CIT-SPECT scan without evidence of dopaminergic deficit (SWEDD), generating a debate about the underlying biological mechanisms. This study investigated differences in clinical features, ^123^I-FP-CIT binding, molecular connectivity, as well as clinical and imaging progression between SWEDD and PD patients.

We included 36 SWEDD, 49 de novo idiopathic PD, and 49 healthy controls with ^123^I-FP-CIT-SPECT from the Parkinson’s Progression Markers Initiative. Clinical and imaging 2-year follow-ups were available for 27 SWEDD and 40 PD. Regional-based and voxel-wise analysis assessed dopaminergic integrity in dorsal and ventral striatal, as well as extrastriatal regions, at baseline and follow-up. Molecular connectivity analyses evaluated dopaminergic pathways. Spatial correlation analyses tested whether ^123^I-FP-CIT-binding alterations would also pertain to the serotoninergic system.

SWEDD and PD patients showed comparable symptoms at baseline, except for hyposmia, which was more severe for PD. PD showed significantly lower striatal and extrastriatal ^123^I-FP-CIT-binding compared to SWEDD and controls. SWEDD exhibited lower binding than controls in striatal regions, insula, and olfactory cortex. Both PD and SWEDD showed extensive altered connectivity of dopaminergic pathways, however, with major impairment in the mesocorticolimbic system for SWEDD. Motor symptoms and dopaminergic deficits worsened after 2 years for PD only.

The limited dopaminergic impairment and its stability over time observed for SWEDD, as well as the presence of extrastriatal ^123^I-FP-CIT binding alterations and prevalent mesocorticolimbic connectivity impairment, suggest other mechanisms contributing to SWEDD pathophysiology.

## Highlights

• PD showed lower ^123^I-FP-CIT-binding than SWEDD at the same symptoms’ severity.
• PD and SWEDD showed altered molecular connectivity but with different patterns.
• Motor symptoms and dopaminergic deficits worsened after 2 years in PD only.
• SWEDD were unlikely to have PD.

## 1 Introduction

Parkinson's disease (PD) is characterized by a prominent neurodegeneration of nigrostriatal pathways. The integrity of the nigrostriatal dopaminergic system can be evaluated with molecular imaging techniques (Politis et al., 2014). Among them, dopamine transporters (DAT) imaging has largely been employed with SPECT using ^123^I-FP-CIT ligand. In PD, DAT imaging displays a striatal binging reduction, predominantly in the striatum contralateral to the clinically most affected side, and has high diagnostic accuracy for distinguishing degenerative parkinsonisms from other conditions, including essential tremor or drug-induced parkinsonism (Marshall et al., 2009). However, a proportion of patients clinically diagnosed with PD have normal DAT scans (labeled as Scan Without Evidence of Dopaminergic Deficit (SWEDD)), generating debates on the underlying biological mechanisms characterizing this subgroup. In large imaging studies for PD, up to 20% have been found to have normal scans (Erro et al., 2016). These patients usually report different clinical features in posture, gait, and tremor, with a major presence of non-motor symptoms, such as orthostatic hypotension, cardiovascular and thermoregulatory dysfunction, daytime sleepiness, cognitive impairment, sleep disturbances, but rarely hyposmia, which is the hallmark of PD (Balestrino et al., 2021). Previous studies on SWEDD populations found minimal or no evidence of disease progression, suggesting that SWEDD subjects are unlikely to have idiopathic PD (Balestrino et al., 2021, Erro et al., 2016, Marek et al., 2014, Marshall et al., 2009, Nicastro et al., 2018). Follow-up studies of SWEDD cases showed that in a significant proportion of these patients, the diagnosis was revised in favor of non-degenerative conditions, including psychogenic parkinsonism, musculoskeletal co-morbidity, dystonia, or essential tremor (Marek et al., 2014, Schneider et al., 2007, Sixel-Döring et al., 2011). It should also be considered that a small proportion of SWEDD subjects, however, may have false-negative imaging because of a misleading SPECT assessment (Nicastro et al., 2016). Until recently and in many studies, the interpretation of ^123^I-FP-CIT SPECT images has been mostly based on visual assessment (Catafau et al., 2004). The visual interpretation, however, leads to difficult-to-classify scans and inter-observer variability that represent serious issues, particularly in patients at an early disease stage. Adding semi-quantitative evaluation to the standard visual assessment improved the recognition of hitherto undetected changes (Nicastro et al., 2018, Söderlund et al., 2013).

Although expert consensus tends to favor abandoning the concept of SWEDD, the debate on its etiology is still ongoing and the mechanisms underpinning clinical symptoms in SWEDD are still unclear. Using a semiquantitative ^123^I-FP-CIT SPECT imaging approach, our study first aims at quantifying presynaptic dopaminergic *in vivo* changes in a large cohort of SWEDD cases, idiopathic PD patients, and healthy subjects from the Parkinson’s Progression Markers Initiative (PPMI). We investigated differences in clinical features, striatal and extrastriatal ^123^I-FP-CIT binding, as well as clinical and imaging progression. Considering the affinity of ^123^I-FP-CIT for transporters involved in different neurotransmission systems (i.e., high-to-low affinity for dopaminergic (DAT) and serotonin (SERT) transporters, respectively) (Booij et al., 2007, Booij and Kemp, 2008), we tested ^123^I-FP-CIT binding in regions innervated by these specific neurotransmitters. Neurotransmission impairment and dysregulation also produce widespread effects on molecular connectivity within distant brain regions, leading to a widespread impairment of brain connectivity in PD (Sala et al., 2017a). Hence, our second aim was to assess interregional association analyses based on molecular data. Finally, given the high affinity of ^123^I-FP-CIT tracer for monoaminergic transporters, we tested whether binding alterations would also pertain to the serotoninergic system by applying spatial correlation analyses. To the best of our knowledge, this is the first study to address molecular connectivity of the dopaminergic systems and the contribution of different monoamine neurotransmitters in SWEDD compared to PD and healthy subjects.

## 2 Methods

### 2.1 Participants

Data used in this study were obtained from the PPMI database (www. ppmi-info.org/data), an international, multiple-site, prospective, longitudinal cohort study. The aims and methodology of PPMI are published elsewhere (Marek et al., 2011, Marek et al., 2018). Study protocols and manuals are available online at https://www.ppmi-info.org/study-design. The institutional review board approved the study at each site, and the participants provided written informed consent.

We included all 49 SWEDD patients from the PPMI who underwent baseline ^123^I-FP-CIT SPECT imaging and T1-weighted MRI within one year. SWEDD subjects have no evidence of dopaminergic deficit in presynaptic dopaminergic scans at visual interpretation and no PD medication within six months from baseline. The visual interpretation for normality was determined by the imaging core of PPMI and was provided to the referring physician. As previous evidence shows that visual interpretation is not sensitive enough to subtle alterations (Nicastro et al., 2018, Söderlund et al., 2013), we semi-quantitatively analyzed all cases (see below) and found 13 cases with significant dopaminergic depletion. We thus excluded these cases for further analyses. This resulted in 36 SWEDD cases, randomly selected 49 de novo idiopathic PD, and 49 healthy controls (HC) with baseline ^123^I-FP-CIT SPECT imaging and T1-weighted MRI within one year. PD patients were drug naïve and subjects with verified genetic mutations known to cause PD (GBA, LRRK2) were excluded.

For all SWEDD and PD subjects, we collected baseline clinical data from the PPMI database (Marek et al., 2011, Marek et al., 2018). 38 SWEDD and 40 PD patients had a clinical assessment at 2-year follow-up. For 36 SWEDD and 39 PD of them, ^123^I-FP-CIT SPECT scans at 2-year follow-up were available.

### 2.2 Clinical and cognitive assessment

Clinical motor assessments of SWEDD and PD samples included Movement Disorders Society-Unified Parkinson’s Disease Rating Scale (MDS- UPDRS) part III and Hoehn and Yahr scales. Subjects were subtyped into tremor dominant (TD), postural instability/gait difficulty (PIGD), and indeterminate (IT) subtypes, using the UPDRS items according to (Stebbins et al., 2013). Clinical non-motor assessments included Epworth Sleepiness Scale and a Rapid Eye Movement (REM) sleep behavior disorder (RBD) questionnaire to assess sleep behavior, Scales for Outcomes in PD-Autonomic (SCOPA-AUT) to assess autonomic function, and the 40-item University of Pennsylvania Smell Identification Test (UPSIT) for olfactory function. Global cognition was assessed with the Montreal Cognitive Assessment (MoCA). Cognitive testing included the Hopkins Verbal Learning Test-Revised (HLVT-R) for memory; Benton Judgment of Line Orientation (JOLO) 15-item version for visuospatial function; and Letter-Number Sequencing (LNS) and semantic fluency for executive abilities-working memory. Neurobehavioral testing included the Geriatric Depression Scale (GDS), State-Trait Anxiety Inventory (STAI), and Questionnaire for Impulsive-Compulsive Disorders (QUIP). All the assessments were also collected at follow-up, except UPSIT.

### 2.3 MRI imaging

The raw T1-weighted MRI imaging data were retrieved from the PPMI collection database. MRI scans’ coordinates were manually set to the anterior commissure as a first step. Volumetric cropped T1-weighted images in native space were segmented into different tissue types to obtain grey and white matter probability maps, using segmentation batch of statistical parametric mapping 12 (SPM12, https://www.fil. ion.ucl.ac.uk/spm/software/spm12). We integrated the grey and white tissue probability maps to create a brain template in native space without non-brain tissue-specific for each patient, using the Image Calculator (ImCalc) sum function in SPM12. Subject-specific subcortical and cortical regions of interest (ROIs) were obtained using the automatic brain structure segmentation of each participant’s MRI scans using the Volbrain platform (Manjón and Coupé 2016). Striatal ROIs, including putamen and caudate nucleus, were subdivided into functional sub-regions, i.e. dorsal-motor and ventral-limbic divisions (Tziortzi et al., 2013). We also considered cortical and subcortical ROIs belonging to the nigrostriatal and the mesocorticolimbic dopaminergic pathways (Boccalini et al., 2022, Sala et al., 2017a). The mesocorticolimbic targets consisted of the anterior and middle cingulate cortices, the olfactory cortex, the insula, the amygdala, hippocampus, and parahippocampal cortex. The nigrostriatal targets consisted of the precentral and postcentral gyri. All regional volumes, extracted with Volbrain, have been compared between PD and SWEDD. The ventral tegmental area, substantia nigra and globus pallidus were not included in the analysis due to the limited spatial resolution of SPECT imaging.

### 2.4 ^123^I-FP-CIT SPECT imaging

We downloaded reconstructed ^123^I-FP-CIT-SPECT imaging data from the PPMI website. Images were acquired on Siemens or General Electric SPECT tomographs, 3–4 h after ^123^I-FP-CIT injection. The imaging protocol for the PPMI scans has been previously documented (Marek et al., 2011, Marek et al., 2018). Pre-processing of SPECT brain images was performed using SPM12, running in MATLAB R2018b Version 9.5.0 (MathWorks Inc., Sherborn, MA, USA). SPECT images were rigidly co-registered to each patient’s brain template in native space. Specific binding ratios (SBR) were calculated as [(target region/reference region)-1] for each ROI. The lateral superior occipital cortex uptake was used as the reference region. In our pipeline the subject-specific ROI approach based on the structure segmentation of MRI scans in each participant was chosen to increase the precision of ^123^I- FP-CIT SBR extraction.

To generate the intensity-normalized parametric images in Montreal Neurologic Institute (MNI) space, T1-weighted MRI brain template of each subject was first normalized to the MNI space using tissue probability maps, then all SPECT images were rigidly aligned to the subject’s respective brain template and normalized to the MNI space using the transformation matrix generated during the registration of the MRI images to standard space. Intensity-normalized parametric images were generated using the lateral superior occipital regions as a reference for each subject throughout the ImCalc function in SPM12. Intensity-normalized images were saved for voxel-wise analyses.

### 2.5 Voxel-wise analysis

Each image was semi-quantitatively analysed by a voxel-wise comparison with HC using SPM12 (Colloby et al., 2004). Each SPECT image was tested at single-subject level for relative dopaminergic depletion by means of a 2-sample *t*-test in comparison with SPECT images of HC. The statistical threshold for the resulting SPM maps was set at a p-value of 0.05, uncorrected for multiple comparisons, considering significant clusters containing more than 100 voxels. This threshold was previously validated for single-subject analyses using ^123^I-FP-CIT SPECT (Colloby et al., 2004).

To complement the ROI-based analysis, a corresponding voxel-based analysis was run at the group level. The ^123^I-FP-CIT SBR parametric images were used in two-sample t-tests in SPM12 to compare SWEDD vs HC and PD vs HC at baseline and, directly SWEDD vs PD at baseline and follow-up. Age was included as a nuisance covariate in the comparison with HC, and age, sex, and UPDRSIII in the direct comparison between SWEDD and PD. Voxel-wise paired t-tests of difference images (baseline and follow-up) within each group (SWEDD and PD) were also run in SPM12. The threshold was set at p = 0.005, family-wise error (FWE)-corrected at the cluster level.

### 2.6 Clinical and ROI-based statistical analysis

T-tests were used to compare demographics and clinical data between SWEDD and PD patients at baseline and follow-up. MANCOVA tests were applied to compare ROI-based SBR imaging data using age, sex, and UPDRSIII as nuisance variables. Paired t-tests of difference images (baseline and follow-up) of clinical and SBR data within each group (SWEDD and PD) were also conducted.

To investigate the differences between SWEDD and PD in cognitive changes over time, we applied linear mixed-effects models for each clinical scale that was used as a dependent variable. Then, applying the same longitudinal model, we examined differences in striatal SBR trajectories between SWEDD and PD.

All analyses were performed using R, version 4.0.2 (https://www.r-project.org/).

### 2.7 Connectivity analysis

Assessment of molecular connectivity between targets of each dopaminergic pathway (nigrostriatal and mesocorticolimbic) was performed via partial correlation analysis computed using MATLAB’s *parcorr* function (Boccalini et al., 2022, Sala et al., 2017b). A subject-by-ROI matrix was created for each group (SWEDD, PD, HC) and contained, for each subject, the SBRs of the specific ROIs for each network. The resulting networks were formed by nodes (ROIs, see Supplementary Material), and by edges, represented by the estimated partial correlation coefficient. Partial correlation coefficients were deemed significant at p < 0.01, uncorrected for multiple comparisons. We applied Fisher’s transformation to the partial correlation coefficients to test whether the strength of each coefficient differed between groups. A Z-test was used to test for significant changes in partial correlation coefficients. All results were set at a statistical threshold of p < 0.01, uncorrected for multiple comparisons.

We calculated the percentage of altered molecular connections in each network for the two groups and we compared the alterations’ percentage between groups for each network through the χ^2^ test.

### 2.8 Spatial correlation analysis

We used the JuSpace toolbox (Dukart et al., 2021) to our dataset to compute Spearman correlation (based on the Neuromorphometrics atlas; adjusted (adj) p-values, N = 1000 permutations) between Z-scores (SWEDD vs HC and PD vs HC) and the DAT (Dukart et al., 2018), and serotonin transporter (SERT) (Hesse et al., 2017) maps, derived from ^123^I-FP-CIT, and ^11^C-3-amino-4-(2-dimethylaminomethyl-phenylsulfanyl)-benzonitrile (^11^C-DASB) data, respectively. The PET/SPECT maps were derived from average group maps of different healthy volunteers and consisted of maps of the binding signal intensity across the whole brain. The data to generate a contrast between patients vs HC were entered as input to be correlated with PET-/SPECT-derived maps. The neuromorphometrics atlas is used to extract mean regional values from the input modalities to be correlated with respective mean values from maps (Dukart et al., 2021).

### 2.9 Data availability

Data are available from the PPMI database (https://www.ppmi-info.org/data).

## 3 Results

13 out of 49 SWEDD showed significant striatal dopaminergic depletion with semi-quantitative analyses (single-subject *t*-test) at baseline. Thus, they were excluded for further analyses. The pattern of dopaminergic depletion of these 13 cases is reported in Supplementary Material (Figure S1). 7 out of these 13 patients (54%) showed dopaminergic depletion at follow-up and 4 out of 13 (31%) showed clinical worsening in motor scales at 2-year follow-up.

Thus, the final SWEDD group included 36 cases. Of these, 27 subjects had a clinical follow-up, and 26 had an imaging follow-up.

**Fig. 1:**
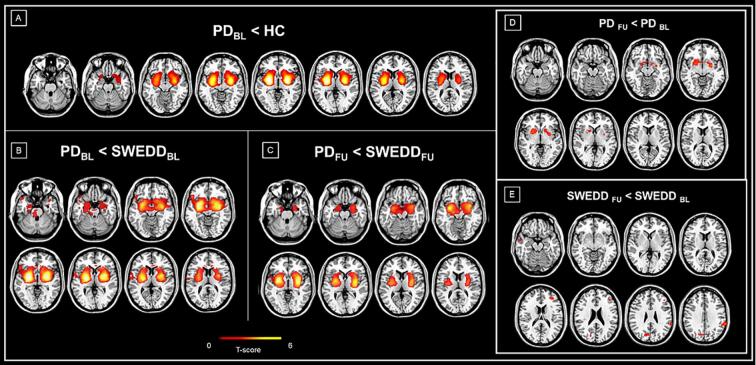
Voxel-wise differences in ^123^I-FP-CIT binding. Panels A shows the distribution of voxel-wise differences in ^123^I-FP-CIT SBR for PD resulting from statistical comparison with HC. Of note, no voxel survived in comparison between SWEDD vs. HC. Panel B shows the results from the direct voxel-wise comparison between SWEDD and PD patients at baseline. Panel C shows the distribution of voxel-wise differences in ^123^I-FP-CIT SBR between PD and SWEDD groups at follow-up. Panels D and E show the results from the direct voxel-wise paired *t*-test between scans at follow-up and baseline in PD (D) and SWEDD (E) groups. The magnitude of the difference is reported by means of t-score. Abbreviations: PD, Parkinson’s disease; SWEDD, scans without evidence of dopaminergic deficit; HC, healthy controls.

SWEDD and PD patients did not differ in any demographic features, namely age (63.4 ± 8.2), sex proportion (37% females), and education (15.2 ± 3.2) (p > 0.05). Clinical features in SWEDD and PD groups at baseline and follow-up are reported in Table 1. ^123^I-FP-CIT imaging results are reported in Fig. 1 and Table 2. A limited proportion of subjects was on antidepressant medication (SSRI/SNRI) (12.2%), equally distributed among PD and SWEDD (*p* = 0.81).

**Table 1:** Clinical features of SWEDD and PD patients at baseline and 2 years follow-up.

	PD at BL	PD at FU	p-value *paired t-test in PD*	SWEDD at BL	SWEDD at FU	p-value *paired t-test in SWEDD*	p-value *SWEDD* vs *PD at BL*	p-value *SWEDD* vs *PD at FU*
**Clinical non-motor assessment**								
MoCA	26.6 (2.11)	25.6 (3.54)	0.059	26.6 (2.60)	26.0 (2.48)	0.164	0.96	0.566
REM Sleep Disorder Questionnaire	4.78 (3.15)	5.47 (3.86)	0.343	4.91 (3.13)	4.59 (3.00)	0.242	0.84	0.298
Epworth Sleepiness Scale	5.65 (3.08)	6.15 (3.06)	0.415	8.57 (4.27)	7.74 (4.52)	0.532	**0.001**	0.118
Scopa-AUT	13.4 (9.33)	16.2 (8.39)	0.063	16.7 (12.1)	17.3 (10.9)	0.988	0.18	0.678
MDS-UPDRS I	5.12 (4.13)	6.97 (4.94)	**0.001**	8.22 (6.30)	9.11 (5.82)	0.264	**0.013**	0.124
**Motor assessment**								
Hoehn and Yahr Staging	1.51 (0.51)	1.77 (0.42)	**0.023**	1.42 (0.50)	1.00 (1.00)	**0.030**	0.4	**<0.001**
MDS-UPDRS II	4.78 (2.73)	7.10 (4.28)	**<0.001**	6.83 (6.33)	8.59 (7.29)	0.064	0.07	0.344
MDS-UPDRS III	18.5 (6.87)	24.3 (10.7)	**<0.001**	15.1 (9.11)	14.4 (11.1)	0.771	0.06	**<0.001**
MDS-UPDRS total score	28.4 (9.91)	38.4 (14.9)	**<0.001**	30.2 (17.3)	45.6 (31.8)	**0.001**	0.59	0.279
**Neurobehavioral assessment**								
GDS	5.20 (1.32)	5.70 (1.77)	0.158	5.60 (1.79)	5.59 (1.76)	0.826	0.27	0.808
QUIP	0.27 (0.57)	1.52 (1.01)	**<0.001**	1.64 (1.13)	2.00 (2.30)	0.386	**<0.001**	0.321
STAI-total score	94.3 (7.71)	93.0 (5.33)	0.345	92.3 (6.67)	91.2 (8.97)	0.865	0.19	0.360
STAI-state score	47.8 (4.67)	47.0 (3.94)	0.612	46.5 (4.50)	45.9 (6.92)	0.807	0.24	0.449
STAI-trait score	46.6 (4.07)	46.0 (3.18)	0.291	45.7 (4.31)	45.4 (3.79)	0.987	0.35	0.464
**Cognitive assessment**								
Benton Judgment of Line Orientation	11.6 (3.37)	11.6 (2.99)	0.851	12.5 (3.00)	11.6 (3.03)	**0.036**	0.22	0.979
Letter number sequencing	11.1 (2.40)	11.3 (3.28)	0.607	9.89 (2.72)	10.7 (3.20)	0.705	**0.034**	0.443
HVLT total immediate recall t- score	45.4 (12.3)	41.7 (10.2)	0.084	44.4 (10.4)	43.2 (12.4)	0.106	0.67	0.615
HVLT delayed recall t-score	42.0 (13.3)	41.2 (13.2)	0.881	41.8 (12.4)	39.4 (13.8)	**0.043**	0.95	0.605
HVLT recognition t-score	42.4 (14.1)	45.0 (12.5)	0.881	36.9 (13.8)	45.2 (9.88)	**0.003**	0.08	0.950
Semantic Fluency t-score	50.4 (9.32)	49.6 (11.0)	0.347	47.9 (10.4)	50.2 (9.10)	0.415	0.26	0.810

Results are reported as mean values and standard deviations (SD, in parentheses).

Abbreviations: SWEDD, scans without evidence of dopaminergic deficit; PD, Parkinson’s disease; y, years; MoCA, Montreal Cognitive Assessment; UPSIT, University of Pennsylvania Smell identification test; MDS-UPDRS, Movement Disorders Society-Unified Parkinson’s Disease Rating Scale; GDS, Geriatric Depression Scale; QUIP, Questionnaire for Impulsive-Compulsive Disorders in Parkinson’s Disease–Rating Scale; STAI, State-Trait Anxiety Inventory; HVLT, Hopkins Verbal Learning Test; BL, baseline; FU, follow-up.

Significant p-values are reported in bold.

**Table 2:** Regional analysis of ^123^I-FP-CIT-SPECT imaging bindings in SWEDD and PD patients at baseline and 2 years follow-up.

	PD at BL	PD at FU	p-value *paired t-test in PD*	SWEDD at BL	SWEDD at FU	p-value *paired t-test in SWEDD*	p-value *SWEDD* vs *PD at BL*	p-value *SWEDD* vs *PD at FU*
Left dorsal caudate	1.34 (0.43)	1.01 (0.44)	**<0.001**	1.59 (0.55)	1.69 (0.58)	0.416	0.065	**<0.001**
Right dorsal caudate	1.44 (0.45)	1.15 (0.47)	**<0.001**	1.77 (0.70)	1.72 (0.61)	0.509	**0.022**	**<0.001**
Left dorsal putamen	1.34 (0.48)	0.98 (0.37)	**<0.001**	2.33 (0.73)	2.29 (0.55)	0.874	**<0.001**	**<0.001**
Right dorsal putamen	1.33 (0.49)	1.03 (0.44)	**<0.001**	2.25 (0.74)	2.34 (0.50)	0.771	**<0.001**	**<0.001**
Left ventral striatum	1.16 (0.44)	0.85 (0.36)	**<0.001**	1.52 (0.54)	1.60 (0.59)	0.382	**<0.001**	**<0.001**
Right ventral striatum	1.22 (0.47)	0.87 (0.37)	**<0.001**	1.50 (0.59)	1.63 (0.60)	0.118	**0.017**	**<0.001**
Left amygdala	0.31 (0.16)	0.33 (0.17)	0.308	0.56 (0.48)	0.48 (0.19)	0.383	**<0.001**	**0.003**
Right amygdala	0.36 (0.20)	0.29 (0.16)	0.173	0.53 (0.50)	0.49 (0.19)	0.680	**0.041**	**<0.001**
Left hippocampus	0.29 (0.11)	0.29 (0.10)	0.900	0.47 (0.35)	0.41 (0.12)	0.441	**0.003**	**<0.001**
Right hippocampus	0.30 (0.12)	0.29 (0.12)	0.854	0.46 (0.33)	0.41 (0.14)	0.550	**0.010**	**<0.001**
Left parahippocampus	0.16 (0.10)	0.17 (0.11)	0.324	0.34 (0.45)	0.25 (0.10)	0.312	**0.026**	**0.013**
Right parahippocampus	0.17 (0.12)	0.15 (0.12)	0.195	0.31 (0.50)	0.24 (0.11)	0.375	0.126	**0.003**
Left insula	0.36 (0.14)	0.29 (0.13)	**<0.001**	0.47 (0.21)	0.50 (0.21)	0.098	0.057	**<0.001**
Right insula	0.34 (0.11)	0.29 (0.10)	**0.013**	0.45 (0.16)	0.48 (0.19)	0.542	**0.010**	**<0.001**
Left olfactory cortex	0.38 (0.22)	0.31 (0.24)	0.084	0.48 (0.24)	0.47 (0.30)	0.749	0.106	**0.022**
Right olfactory cortex	0.39 (0.24)	0.30 (0.23)	**0.007**	0.48 (0.28)	0.44 (0.27)	0.924	0.165	**0.027**
Left anterior cingulate	0.21 (0.10)	0.17 (0.11)	0.035	0.18 (0.15)	0.18 (0.12)	0.613	0.316	0.657
Right anterior cingulate	0.22 (0.10)	0.18 (0.11)	0.007	0.17 (0.14)	0.20 (0.13)	0.112	**0.034**	0.492
Left middle cingulate	0.25 (0.12)	0.21 (0.12)	0.054	0.28 (0.11)	0.29 (0.13)	0.235	0.436	**0.012**
Right middle cingulate	0.25 (0.12)	0.23 (0.12)	0.293	0.29 (0.12)	0.30 (0.15)	0.666	0.224	**0.014**
Left postcentral gyrus	0.07 (0.07)	0.04 (0.06)	0.098	0.05 (0.06)	0.05 (0.06)	0.396	0.568	0.505
Right postcentral gyrus	0.06 (0.05)	0.05 (0.07)	0.438	0.06 (0.07)	0.06 (0.07)	0.432	0.982	0.927
Right precentral gyrus	0.16 (0.12)	0.15 (0.12)	0.561	0.17 (0.12)	0.16 (0.11)	0.941	0.623	0.767
Left precentral gyrus	0.15 (0.11)	0.14 (0.11)	0.733	0.16 (0.10)	0.16 (0.11)	0.487	0.743	0.588

Results are reported as mean values and standard deviations (SD, in parentheses).

Abbreviations: SWEDD, scans without evidence of dopaminergic deficit; PD, Parkinson’s disease; BL, baseline; FU, follow-up.

Significant p-values are reported in bold.

### 3.1 Clinical differences

**Baseline** – PD and SWEDD patients did not significantly differ in motor impairments (Table 1 and Figure S2) or subtypes (p = 0.84). PD patients presented significantly worse smell impairment as measured by UPSIT than SWEDD (p < 0.001). SWEDD patients presented higher MDS-UPDRS part I scores, indicating more, even if mild, nonmotor deficits of daily living compared to PD. PD and SWEDD significantly differed in the Epworth Sleepiness Scale and QUIP (measuring impulsive-compulsive behaviors), but both groups presented average scores within normal values.

**Follow-up** – PD and SWEDD patients significantly differed in the motor assessment, with PD showing higher Hoehn and Yahr stages and MDS-UPDRS part III scores compared to SWEDD (Table 1). Paired *t*-test results showed a worsening from baseline to follow-up in all motor scales and QUIP (Table 1). SWEDD patients presented a worsening in memory (HVLT), and visuospatial skills (JOLO) at 2 year-follow-up. MDS-UPDRS total score increased for SWEDD and PD. Linear mixed effect models indicated that PD group showed a significantly faster decline over time on MDS-UPDRS part III (p < 0.001) and MDS-UPDRS total score (p = 0.04) compared to SWEDD (Figure S2).

## 4 ^123^I-FP-CIT SPECT imaging differences

**Baseline** –ROI-based results showed more significant SBR decreases for PD patients compared to SWEDD in several striatal (ventral striatum, dorsal caudate, and dorsal putamen) and extrastriatal (amygdala, hippocampus, parahippocampus, insula) ROIs within the nigrostriatal and mesocorticolimbic dopaminergic systems (Table 2). SWEDD showed lower binding than PD only in the ACC as shown by ROI-based results (Table 2). Voxel-wise analysis did not find any difference between SWEDD and HC, whereas PD exhibited lower binding in the striatal and limbic extrastriatal regions in comparison with HC (Fig. 1**A**) and SWEDD (Fig. 1**B**). Notably, at variance with voxel-wise analyses, ROI-based results showed lower SBR in SWEDD patients compared to HC in striatal regions, insula, hippocampus, and olfactory cortex, without reaching the same severity of PD (Table S1). When we compared the regional volumes between PD and SWEDD, there were no areas in which the PD group had a significantly lower volume than SWEDD (Table S2).

**Follow-up** – PD patients showed lower SBR than SWEDD in all striatal and many extrastriatal ROIs, namely the amygdala, insula, hippocampus, parahippocampus, olfactory cortex, and middle cingulate (Table 2). These differences were confirmed in voxel-wise analysis (Fig. 1**C**). No SBR decreases were observed over time in any ROIs for SWEDD patients, whereas SBR significantly decreased for PD at follow-up in the striatum, insula, and right olfactory cortex (Table 2**,** Fig. 1**D and 1E**). Linear mixed effect models indicated that the PD group showed a faster decline over time in striatal dopaminergic denervation (p < 0.05) compared to SWEDD.

**Neurotransmitter mapping** - Extrastriatal mapping of ^123^I-FP-CIT binding alterations in other monoaminergic systems was assessed. We observed a significant spatial correlation between DAT and SERT alterations (p < 0.001) for PD compared to HC (z-scores). However, we found only a trend for significance in the correlation between ^123^I-FP- CIT alterations and serotonergic map in SWEDD (p = 0.059). Figure S3 shows Fisher’s z-transformed correlation coefficients with respective neurotransmitter maps for each subject (individual points) and contrast.

### 4.1 Molecular connectivity differences

**Fig. 2:**
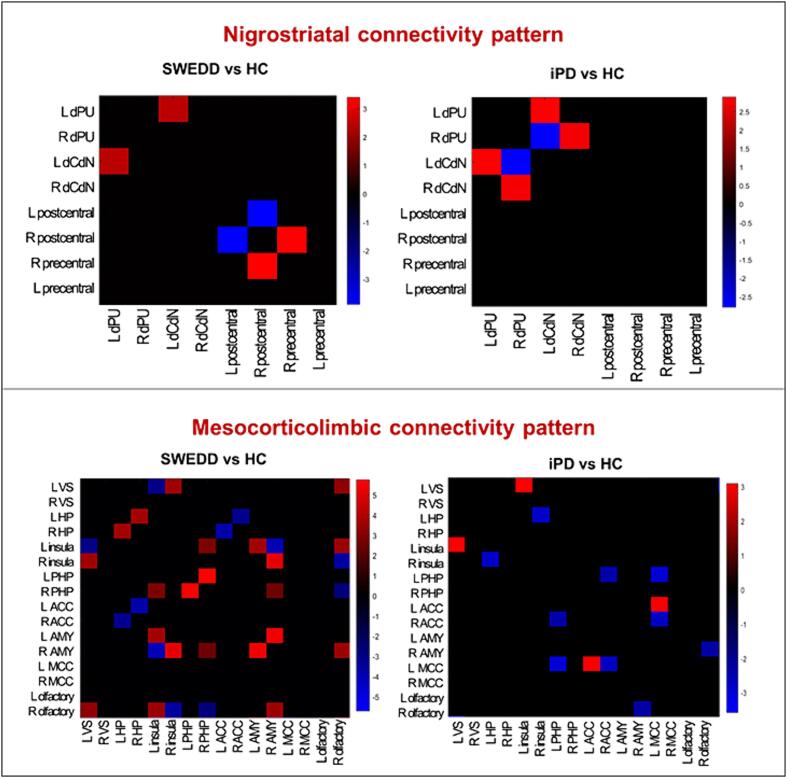
Dopaminergic connectivity results in SWEDD and PD groups. The matrices represent the significant differences obtained when comparing partial correlation coefficients between SWEDD vs HC, and PD vs HC in the dopaminergic networks. The color bar displays the Z scores’ values to compare partial correlation coefficients’ strengths. Altered connections are presented: in red, the increased and in blue, the decreased connections compared with HC. Abbreviations: PD, idiopathic Parkinson’s disease; SWEDD, scans without evidence of dopaminergic deficits; HC, healthy controls; L, left; R, right; dCdN, dorsal caudate nucleus; dPU, dorsal putamen, VS, ventral striatum, HIP, hippocampus; PHP, parahippocampus; AMY, amygdala; ACC, anterior cingulate cortex, MCC, middle cingulate cortex.

**Nigrostriatal dopaminergic system** – Several connectivity alterations affected both SWEDD and PD nigrostriatal system, with 12.5% of altered connections for both groups compared to HC. However, these alterations were characterized by different connectivity patterns (Fig. 2). PD connectivity pattern showed only short-distance local subcortical alterations within the striatum. SWEDD patients also showed short-distance cortical alterations between the precentral and postcentral gyri, specifically connectivity increases characterizing ipsilateral regions and connectivity decreases contralateral ones.

**Mesocorticolimbic dopaminergic system** – SWEDD and PD patients showed 15.2% and 6.2% altered connections, respectively, compared to HC (Fig. 2). 64.7 % of the altered connections in SWEDD were represented by increases compared to HC, whereas in PD most of the alterations (71.5 %) were represented by decreased in connectivity. For SWEDD, the alterations involved many extrastriatal regions, mostly the insula, hippocampus, and amygdala as well as the right olfactory cortex. For PD, we observed altered connectivity between the ventral striatum and insula, between the insula and hippocampus, in addition to ACC and MCC. SWEDD showed more connectivity alterations compared to PD (χ2 = 4.30, p = 0.03).

## 5 Discussion

This study demonstrated that ^123^I-FP-CIT SPECT imaging can reliably reveal distinct dopaminergic alterations in early PD and SWEDD at baseline and their progression at a 2-year follow-up. Our baseline results first indicated that SWEDD had significantly less striatal and extrastriatal dopaminergic depletion than PD, while clinical severity is similar. Longitudinal imaging results further supported that the majority of SWEDD were unlikely to have PD since they did not worsen with regards to motor symptoms and neither in striatal nor extrastriatal dopaminergic depletion at follow-up. On the other hand, for the first time, we found extensive altered connectivity in dopaminergic systems, however with different patterns in PD and SWEDD. The latter group was characterized by more severe extrastriatal cortical connectivity impairment. When we mapped the ^123^I-FP-CIT binding alterations with monoaminergic neurotransmitter topography in both groups, we found significant spatial associations with dopaminergic and serotonergic systems only in PD, and a trend with the serotonergic one in SWEDD.

From a clinical standpoint, the two groups were comparable in terms of motor and cognitive symptoms at baseline (Table 1). PD patients presented significantly more severe smell impairments than SWEDD, that instead showed more pronounced non-motor symptoms (even if mild). Among non-motor symptoms, hyposmia has been widely recognized as an early discriminative sign of PD (Sprenger et al., 2015, Taylor et al., 2016). Our results further supported that hyposmia might be particularly useful to differentiate the two groups. Previous literature reported discordant results regarding differences in non-motor symptoms, with a minor, comparable, or greater severity in SWEDD subjects (Balestrino et al., 2021). The heterogeneous clinical phenotypes encompassed by SWEDD subjects may at least partially explain the high degree of non-motor symptoms burden found here in SWEDD.

Even if the motor, autonomic, and cognitive symptoms similarly characterized both groups at baseline, worsening at 2-year follow-up occurred only in PD (Table 1**)**. PD patients worsened in all the MDS-UPDRS scales over time, while SWEDD only showed a slight worsening in memory and visuospatial skills and MDS-UPDRS total score, mostly driven by non-motor scores. A lack of clinical progression in SWEDD strongly argues against a diagnosis of PD (and a neurodegenerative condition in general) (Balestrino et al., 2021, Lee, et al., 2021, Marshall et al., 2009, Nicastro et al., 2020). Notably, previous studies already demonstrated that for many SWEDD cases, the clinical diagnosis was revised in favor of dystonic tremor, or other alternative diagnoses included vascular parkinsonism, essential/atypical/monosymptomatic tremor, frontotemporal lobar degeneration with motor signs, functional (psychogenic) parkinsonism, drug-induced parkinsonism, and mild parkinsonian signs attributed to aging (see (Balestrino et al., 2021) for a review).

From the imaging side, we applied two different approaches, namely a voxel-based and a subject-specific ROI-based approach based on the structure segmentation of MRI scans to increase the SBR precision. PD subjects showed significantly lower binding in both striatal and extrastriatal regions compared with SWEDD and controls (Table 2, Fig. 1**A and 1B**). Importantly, given the limitations of the visual interpretation approach (Batla et al., 2014, Nicastro et al., 2018, Söderlund et al., 2013), SWEDD were first semi-quantitatively evaluated by applying a voxel-wise comparison with HC. 26% of scans initially reported as visually negative were found abnormal (Figure S1) and thus excluded from the analyses. Of note, SPECT misinterpretation has emerged as an important contributor to the SWEDD population since a proportion of patients might have instead a degenerative condition and a better diagnosis can be reached by combining visual rating with semi-quantification (Nicastro et al., 2016, Nicastro et al., 2018). For instance, among the conditions underlying SWEDD, essential tremor could be a neurodegenerative disorder since some patients were found to have a subtle striatal dopaminergic deficit with uptake value below the control level and above the level seen in PD (Isaias et al., 2008, Yoo et al., 2023).

To the best of our knowledge, only one previous study investigated extrastriatal ^123^I-FP-CIT binding in SWEDD demonstrating preserved values comparably to HC in SWEDD whereas PD subjects showed reduced bindings in the insula and thalamus (Nicastro,Burkhard,and Garibotto, 2020). Our results showed lower binding in the insula and thalamus but also in the hippocampus and parahippocampus for PD compared to SWEDD (Fig. 1**B** and Table 2). These extrastriatal regions harbor a variable proportion of SERT, for which ^123^I- FP-CIT has a moderate affinity (Koch et al., 2014), confirming a serotoninergic loss in PD. Serotonergic binding as assessed by DASB was diffusely reduced in the striatum, brainstem, and multiple cortical areas in PD (Politis et al., 2010). SWEDD also had lower binding in the ACC, whose serotonergic dysfunction has a prominent role in apathy, depression, and anxiety (Hsam and Kohl, 2023, Maillet et al., 2021). Moreover, SWEDD showed a higher hippocampal SBR compared to HC (Table S1) suggesting a possible altered serotoninergic transmission. The hypothesis of compensatory upregulation of serotonergic nerve terminals has been advanced to explain the relative preservation of ^18^F-dopa uptake in a genetic case of PD (Wile et al., 2016). However, the possible contribution of the serotoninergic system, as well as other neurotransmitter systems, to SWEDD symptomatology has never been directly explored. When we applied spatial association analysis with specific neurotransmitter maps (Dukart et al., 2021), we found ^123^I-FP-CIT binding alterations associated with SERT in PD and only a trend for significance in SWEDD (Figure S3), likely because of the limited sample size.

Our longitudinal imaging results showed that striatal and extrastriatal deficit worsened at 2-year follow-up in PD but not in SWEDD (Fig. 1**D** and Table 2), which is in line with previous DAT studies focusing on striatal binding (Batla et al., 2014, Lee, et al., 2021, Marek et al., 2014, Marshall et al., 2006). The lack of dopaminergic depletion worsening in SWEDD suggests that these subjects suffer from other conditions.

To assess the neural mechanisms underlying SWEDD, we explored for the first time the molecular connectivity in the two main dopaminergic systems, finding an extended cortical connectivity impairment characterizing SWEDD. PD’s nigrostriatal system was primarily characterized by a loss of interconnections within the basal ganglia, whereas SWEDD also showed cortical alterations. The group differences were more marked in the mesocorticolimbic system, with SWEDD showing greater connectivity alterations than PD (Fig. 2). These alterations involved the ventral striatum and many extrastriatal regions innervated also by other neurotransmission systems. Most of the alterations were represented by increases in connectivity in SWEDD rather than decreases, as occurred in PD. Connectivity decreases might be indicative of selective denervation from the neurotransmitter nuclei projecting to the target regions, whereas interpreting the significance of connectivity increases is particularly complex. Increased connectivity might be indicative of a compensatory process with the recruitment of brain regions that are still functional. But it can also represent a form of maladaptive functional reorganization where the proper connections of healthy brains are replaced by a widespread dysfunctional connectivity pattern (Hillary and Grafman, 2017). Moreover, the presence of cortical and extrastriatal connectivity alterations might suggest other possible neurotransmission abnormalities contributing to SWEDD symptomatology. The consensus about SWEDD is that it represents a variegated group of different conditions mimicking PD, including functional tremor, essential tremor, or dystonia. Interestingly, altered connectivity in limbic areas has been observed in functional movement disorders (Morgante,Edwards,and Espay, 2013), while we did not find such evidence for other conditions. Future molecular imaging studies using specific radiotracers are needed to understand the pathogenetic mechanisms and, thus, to address appropriate therapeutic options.

As a possible limitation of the study, we acknowledge that we did not correct for the partial volume effect (PVE). Our choice was based on the lack of absolute consensus on all the available methods, as PVE correction might introduce random noise and unpredictably alter regional SBR (Kanel et al., 2023), and also considering that our target cohort including early-stage disease and healthy controls that have presumably limited atrophy and that the used radiotracer is characterized by high affinity and specificity for DAT.

Together with previous clinical and ^123^I- FP-CIT PET results, the present findings highlight the distinct clinical and molecular trajectories of PD and SWEDD subjects. SWEDD subjects are characterized by prominent non-motor symptoms, absence of hyposmia, and generally preserved dopaminergic binding but altered cortical connectivity. One major question will remain to have a final diagnosis for SWEDD, as they represent mostly a variegated group of non-degenerative conditions mimicking PD. Dopaminergic studies on each of these conditions would be useful to address this issue. Moreover, further studies are needed to explore other semiquantitative approaches including commercial or non-commercial software typically used for classification of DaT SPECT in the clinical setting, to define the best methods and their consistency in classifying problematic scans.

## CRediT authorship contribution statement

**Cecilia Boccalini:** Conceptualization, Data curation, Formal analysis, Investigation, Methodology, Writing – original draft. **Nicolas Nicastro:** Data curation, Investigation, Writing – review & editing. **Daniela Perani:** Conceptualization, Investigation, Writing – review & editing. **Valentina Garibotto:** Conceptualization, Data curation, Investigation, Supervision, Writing – review & editing.

## Declaration of competing interest

The authors declare that they have no known competing financial interests or personal relationships that could have appeared to influence the work reported in this paper.

## Data Availability

Data are available from the PPMI database (www.ppmi-info.org/data).

## Appendix A

Parkinson’s Progression Marker Initiative Authors—Steering committee: Kenneth Marek, MD1; Andrew Siderowf, MD, MSCE2; John Seibyl, MD1; Christopher Coffey, PhD3; Caroline Tanner, MD, PhD4; Duygu Tosun- Turgut, PhD4; Tanya Simuni, MD5; Leslie M. Shaw, PhD6; John Q. Trojanowski, MD, PhD2; Andrew Singleton, PhD7; Karl Kieburtz, MD, MPH9; Arthur Toga, PhD8; Brit Mollenhauer, MD9; Douglas Galasko, MD10; Lana M. Chahine, MD11; Werner Poewe, MD12; Tatiana Foroud, PhD 13; Kathleen Poston, MD, MS14; Susan Bressman, MD15 Alyssa Reimer16; Vanessa Arnedo16; Adrienne Clark16; Mark Frasier, PhD16; Catherine Kopil, PhD16; Sohini Chowdhury16; Todd Sherer, PhD.16 Study cores: leadership core—Kenneth Marek, MD1; Nichole Daegele1; clinical coordination core—Cynthia Casaceli, MBA,17 Ray Dorsey, MD, MBA,17 Renee Wilson,17 Sugi Mahes17; imaging core—John Seibyl, MD,1 Christina Salerno1; statistics core—Christopher Coffey, PhD,3 Chelsea Caspell-Garcia3; bioinformatics core—Arthur Toga, PhD,8 Karen Crawford8; biorepository—Tatiana Foroud, PhD,13 Paola Casa- lin,18 Giulia Malferrari,18 Mali Gani Weisz,19 Avi Orr-Urtreger, MD, PhD19; bioanalytics core—John Trojanowski, MD, PhD,2 Leslie Shaw, PhD2; genetics core—Andrew Singleton, PhD7; genetics coordination core—Tatiana Foroud, PhD13; pathology core—Tatiana Foroud, PhD,13 Thomas Montine, MD, PhD14; wearables core—Tatiana Foroud, PhD13; advanced analytics core—Chris Baglieri,65 Amanda Christini, MD.65 Site investigators: David Russell, MD, PhD1; Caroline Tanner, MD4; Tanya Simuni, MD5; Nabila Dahodwala, MD2; Brit Mollenhauer MD9; Douglas Galasko, MD10; Werner Poewe, MD12; Nir Giladi, MD19; Stewart Factor, DO20; Penelope Hogarth, MD21; David Standaert, MD, PhD22; Robert Hauser, MD, MBA23; Joseph Jankovic, MD24; Marie Saint-Hilaire, MD25; Irene Richard, MD26; David Shprecher, DO27; Hubert Fernandez, MD28; Katrina Brockmann, MD29; Liana Rosenthal, MD30; Paolo Barone, MD, PhD31; Alberto Espay, MD, MSc32; Dominic Rowe BSc, MBBS33; Karen Marder, MD, MPH34; Anthony Santiago, MD35; Susan Bressman, MD36; Shu-Ching Hu, MD, PhD37; Stuart Isaacson, MD38; Jean-Christophe Corvol, MD39; Javiar Ruiz Martinez, MD40; Eduardo Tolosa, MD41; Yen Tai, MD42; Marios Politis, MD, PhD.43 Coordinators: Debra Smejdir1; Linda Rees, MPH1; Karen Wil- liams3; Farah Kausar4; Karen Williams5; Whitney Richardson2; Diana Willeke9; Shawnees Peacock10; Barbara Sommerfeld, RN, MSN20; Ali- son Freed21; Katrina Wakeman22; Courtney Blair, MA23; Stephanie Guthrie, MSN24; Leigh Harrell23; Christine Hunter, RN24; Cathi-Ann Thomas, RN, MS25; Raymond James, RN25; Grace Zimmerman26; Victoria Brown27; Jennifer Mule BS28; Ella Hilt29; Kori Ribb30; Susan Ainscough31; Misty Wethington32; Madelaine Ranola33; Helen Mejia Santana34; Juliana Moreno35; Deborah Raymond36; Krista Speketer37; Lisbeth Carvajal38; Stephanie Carvalo39; Ioana Croitoru40; Alicia Garrido, MD41; Laura Marie Payne, BSC.42 Industry and scientific advisory board: Veena Viswanth, PhD44; Lawrence Severt, PhD44; Maurizio Facheris, MD45; Holly Soares, PhD45; Mark A. Mintun, MD46; Jesse Cedarbaum, MD47; Peggy Taylor, ScD48; Kevin Biglan, MD49; Emily Vandenbroucke, PhD50; Zulfiqar Haider Sheikh50; Baris Bin- gol51; Tanya Fischer, MD, PhD52; Pablo Sardi, PhD52; Remi Forrat52; Alastair Reith, PhD53; Jan Egebjerg, PhD54; Gabrielle Ahlberg Hill- ert54; Barbara Saba, MD55; Chris Min, MD, PhD56; Robert Umek, PhD57; Joe Mather58; Susan De Santi, PhD59; Anke Post, PhD60; Frank Boess, PhD60; Kirsten Taylor60; Igor Grachev, MD, PhD61; Andreja Avbersek, MD62; Pierandrea Muglia, MD62; Kaplana Merchant, PhD63; Johannes Tauscher, MD64 Affiliations: 1 Institute for Neurodegenerative Disorders, New Haven,

CT; 2 University of Pennsylvania, Philadelphia, PA; 3 University of Iowa, Iowa City, IA; 4 University of California, San Francisco, CA; 5 North- western University, Chicago, IL; 7 National Institute on Aging, NIH, Bethesda, MD; 8 Laboratory of Neuroimaging (LONI), University of Southern California, Los Angeles, CA; 9 Paracelsus-Elena Klinik, Kassel, Germany; 10 University of California, San Diego, CA; 11 University of Pittsburgh, Pittsburgh, PA; 12 Innsbruck Medical University, Innsbruck, Austria; 13 Indiana University, Indianapolis, IN; 14 Stanford University, Stanford, California; 15 Mount Sinai, New York, NY; 16 The Michael J. Fox Foundation for Parkinson’s Research, New York, NY; 17 Clinical Trials Coordination Center, University of Rochester, Rochester, NY; 18 BioRep, Milan, Italy; 19 Tel Aviv Medical Center, Tel Aviv, Israel; 20 Emory University of Medicine, Atlanta, GA; 21 Oregon Health and Science University, Portland, OR; 22 University of Alabama at Birmingham, Birmingham, AL; 23 University of South Florida, Tampa, FL; 24 Baylor College of Medicine, Houston, TX; 25 Boston University, Boston, MA; 26 University of Rochester, Rochester, NY; 27 Banner Research Institute, Sun City, AZ; 28 Cleveland Clinic, Cleveland, OH; 29 University of Tuebingen, Tuebingen, Germany; 30 Johns Hopkins University, Balti- more, MD; 31 University of Salerno, Salerno, Italy; 32 University of Cincinnati, Cincinnati, OH; 33 Macquarie University, Sydney Australia; 34 Columbia University, New York, NY; 35 The Parkinson’s Institute, Sunnyvale, CA; 36 Beth Israel Medical Center, New York, NY; 37 Uni- versity of Washington, Seattle, WA; 38 Parkinson’s Disease and Move- ment Disorders Center of Boca Raton, Boca Raton, FL; 39 Hospital Pitie- Salpetriere, Paris, France; 40 Hospital Donostia, San Sebastian, Spain; 41 Hospital Clinic de Barcelona, Barcelona, Spain; 42 Imperial College London, London, United Kingdom; 43 King’s College London, London, United Kingdom; 44 Allergan, Dublin, Ireland; 45 Abbvie, North Chi- cago, IL; 46 Avid Radiopharmaceuticals, Inc., Philadelphia, PA; 47 Biogen Idec, Cambridge, MA; 48 BioLegend, Dedham, MA; 49 Eli Lilly and Company, Indianapolis, IN; 50 GE Healthcare, Princeton, NJ; 51 Genentech, San Francisco, CA; 52 Genzyme Sanofi, Cambridge, MA; 53 GlaxoSmithKline, Brentford, United Kingdom; 54H. Lundbeck A/S, Copenhagen, Denmark; 55 Institut de Recherches Internationales Serv- ier, Neuilly-sur-Seine, France; 56 Merck and Co., Kenilworth, NJ; 57 Meso Scale Diagnostics, Rockville, MD; 58 Pfizer Inc., Cambridge, MA; 59 Piramal Group, Mumbai, India; 60F. Hoffmann-La Roche Limited, Basel, Switzerland; 61 Teva Pharmaceutical Industries, Petah Tikva, Israel; 62 UCB Pharma, Brussel, Belgium; 63 TransThera Consulting, Portland, OR; 64 Takeda, Osaka, Japan; 65 Blackfynn, Philadelphia, PA.
