# Supplementary material for: Distinctive clinical and imaging trajectories in SWEDD and Parkinson’s disease patients
Source: Neuroimage Clin. 2024 Mar 14;42:103592. doi: 10.1016/j.nicl.2024.103592 (PMC10958480; doi:10.1016/j.nicl.2024.103592)
Supplement: Supplementary data 1 [file mmc1.docx]

**Supplementary Materials**

**Connectivity analysis**

ROIs were selected based only on the cortical targets that showed significant tracer binding in HC. This resulted in a pool of N=8 ROIs belonging to the nigrostriatal pathway (left/right (L/R) dorsal caudate nucleus, L/R dorsal putamen, and L/R precentral and postcentral gyri), and N=16 ROIs belonging to the mesocorticolimbic pathway (L/R ventral striatum, L/R hippocampus, L/R amygdala, L/R insula, L/R olfactory cortex, L/R parahippocampus, L/R anterior cingulate cortex, and L/R middle cingulate cortex).

**Table S1 Regional analysis of ^123^I-FP-CIT-SPECT imaging bindings in the whole sample at baseline**

|  | **HC** | **iPD** | **SWEDD** | **p-value** | **p-value** | **p-value** |
| --- | --- | --- | --- | --- | --- | --- |
|  |  |  |  | ***iPD vs. HC*** | ***SWEDD vs. HC*** | ***iPD vs. SWEDD*** |
| Left dorsal caudate | 2.17 (0.68) | 1.34 (0.43) | 1.59 (0.55) | **0.000** | **0.002** | 0.065 |
| Right dorsal caudate | 2.35 (0.82) | 1.44 (0.45) | 1.77 (0.70) | **0.000** | **0.002** | **0.022** |
| Left dorsal putamen | 3.09 (0.66) | 1.34 (0.48) | 2.33 (0.73) | **0.006** | **0.002** | **<0.001** |
| Right dorsal putamen | 3.00 (0.67) | 1.33 (0.49) | 2.25 (0.74) | **0.000** | **0.001** | **<0.001** |
| Left ventral striatum | 2.13 (0.52) | 1.16 (0.44) | 1.52 (0.54) | **0.000** | **0.001** | **<0.001** |
| Right ventral striatum | 2.12 (0.46) | 1.22 (0.47) | 1.50 (0.59) | **0.000** | **0.001** | **0.017** |
| Left amygdala | 0.44 (0.30) | 0.31 (0.16) | 0.56 (0.48) | 0.291 | 0.148 | **<0.001** |
| Right amygdala | 0.44 (0.31) | 0.36 (0.20) | 0.53 (0.50) | 0.482 | 0.235 | **0.041** |
| Left hippocampus | 0.33 (0.20) | 0.29 (0.11) | 0.47 (0.35) | 0.771 | **0.023** | **0.003** |
| Right hippocampus | 0.33 (0.18) | 0.30 (0.12) | 0.46 (0.33) | 0.800 | **0.037** | **0.010** |
| Left parahippocampus | 0.21 (0.18) | 0.16 (0.10) | 0.34 (0.45) | 0.686 | 0.074 | 0.026 |
| Right parahippocampus | 0.22 (0.16) | 0.17 (0.12) | 0.31 (0.50) | 0.721 | 0.214 | 0.126 |
| Left insula | 0.70 (0.22) | 0.36 (0.14) | 0.47 (0.21) | **0.000** | **0.004** | 0.057 |
| Right insula | 0.58 (0.20) | 0.34 (0.11) | 0.45 (0.16) | **0.001** | 0.066 | **0.010** |
| Left olfactory cortex | 0.86 (0.44) | 0.38 (0.22) | 0.48 (0.24) | **0.000** | **0.000** | 0.106 |
| Right olfactory cortex | 0.84 (0.44) | 0.39 (0.24) | 0.48 (0.28) | **0.000** | **0.000** | 0.165 |
| Left anterior cingulate | 0.18 (0.11) | 0.21 (0.10) | 0.18 (0.15) | 0.495 | 0.822 | 0.316 |
| Right anterior cingulate | 0.21 (0.12) | 0.22 (0.10) | 0.17 (0.14) | 0.668 | 0.158 | **0.034** |
| Left middle cingulate | 0.24 (0.13) | 0.25 (0.12) | 0.28 (0.11) | 0.287 | 0.119 | 0.436 |
| Right middle cingulate | 0.25 (0.12) | 0.25 (0.12) | 0.29 (0.12) | 0.271 | 0.054 | 0.224 |
| Left postcentral gyrus | 0.12 (0.08) | 0.07 (0.07) | 0.05 (0.06) | **0.050** | **0.009** | 0.568 |
| Right postcentral gyrus | 0.07 (0.08) | 0.06 (0.05) | 0.06 (0.07) | 0.995 | 0.949 | 0.982 |
| Right precentral gyrus | 0.14 (0.09) | 0.16 (0.12) | 0.17 (0.12) | 0.963 | 0.717 | 0.623 |
| Left precentral gyrus | 0.08 (0.09) | 0.15 (0.11) | 0.16 (0.10) | **0.023** | **0.004** | 0.743 |
| *Results are reported as mean values and standard deviations (SD, in parentheses).*  *Abbreviations: SWEDD,* *scans without evidence of dopaminergic deficit; iPD, idiopathic Parkinson’s disease; BL, baseline; FU, follow-up.*  *Significant p-values are reported in bold.* | | | | | | |

**Table S2 Regional differences in volumes (cm^3^) obtained using Volbrain platform between PD and SWEDD at baseline**

|  | **iPD** | **SWEDD** | **p-value** |
| --- | --- | --- | --- |
|  |  |  |  |
| Grey Matter | 665 (61.9) | 639 (81.0) | 0.096 |
| Subcortical Grey Matter | 40.7 (3.19) | 39.3 (6.03) | 0.218 |
| Cortical Grey Matter | 518 (50.8) | 494 (66.0) | 0.064 |
| Left caudate | 3.36 (0.34) | 3.28 (0.65) | 0.502 |
| Right caudate | 3.36 (0.43) | 3.34 (0.71) | 0.828 |
| Left putamen | 4.06 (0.48) | 3.82 (0.99) | 0.185 |
| Right putamen | 4.03 (0.51) | 3.82 (0.95) | 0.231 |
| Left amygdala | 1.03 (0.12) | 0.97 (0.22) | 0.141 |
| Right amygdala | 1.02 (0.11) | 0.97 (0.21) | 0.158 |
| Right accumbens | 0.29 (0.06) | 0.28 (0.08) | 0.382 |
| Left accumbens | 0.33 (0.06) | 0.31 (0.08) | 0.126 |
| Left hippocampus | 4.04 (0.44) | 3.97 (0.61) | 0.511 |
| Right hippocampus | 4.12 (0.40) | 4.05 (0.66) | 0.569 |
| Right insula | 14.9 (1.63) | 13.8 (2.13) | 0.013 |
| Left insula | 14.3 (1.46) | 13.3 (2.05) | **0.022** |
| Right anterior cingulate | 5.13 (0.95) | 4.82 (0.95) | 0.128 |
| Left anterior cingulate | 5.72 (0.86) | 5.27 (1.15) | **0.044** |
| Right middle cingulate | 5.18 (0.75) | 4.75 (0.92) | **0.020** |
| Left middle cingulate | 5.20 (0.69) | 4.81 (0.90) | **0.031** |
| Right parahippocampus | 2.88 (0.36) | 2.84 (0.48) | 0.673 |
| Left parahippocampus | 3.14 (0.40) | 3.11 (0.51) | 0.780 |
| Right precentral gyrus | 12.4 (1.42) | 11.8 (1.73) | 0.088 |
| Left precentral gyrus | 12.2 (1.43) | 11.8 (1.65) | 0.239 |
| Right postcentral gyrus | 8.98 (1.24) | 8.70 (1.15) | 0.263 |
| Left postcentral gyrus | 9.42 (1.24) | 9.01 (1.43) | 0.160 |
| *Results are reported as mean values and standard deviations (SD, in parentheses).*  *Abbreviations: SWEDD,* *scans without evidence of dopaminergic deficit; iPD, idiopathic Parkinson’s disease; Significant p-values are reported in bold.* | | | |

**Supplementary figures**


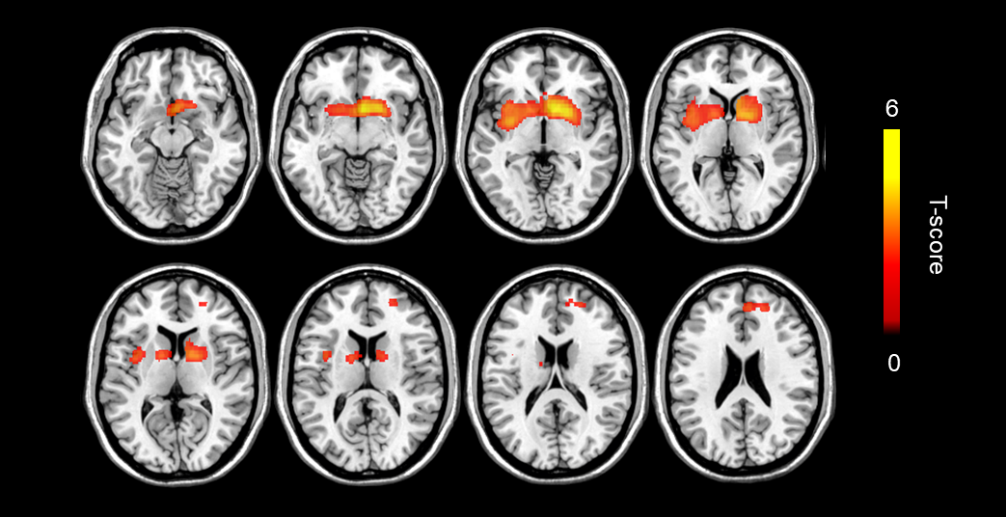


**Figure S1 Voxel-wise differences in ^123^I-FP-CIT binding between excluded SWEDD (n=13) and HC.** The figure shows the distribution of voxel-wise differences in ^123^I-FP-CIT SBR for 13 SWEDD patients (excluded because of the presence of striatal dopaminergic depletion at single-subject analysis) resulting from statistical comparison with HC at baseline. Here, the patients show striatal dopaminergic depletion compared to HC at the group level. The magnitude of the difference is reported by means of t-score.


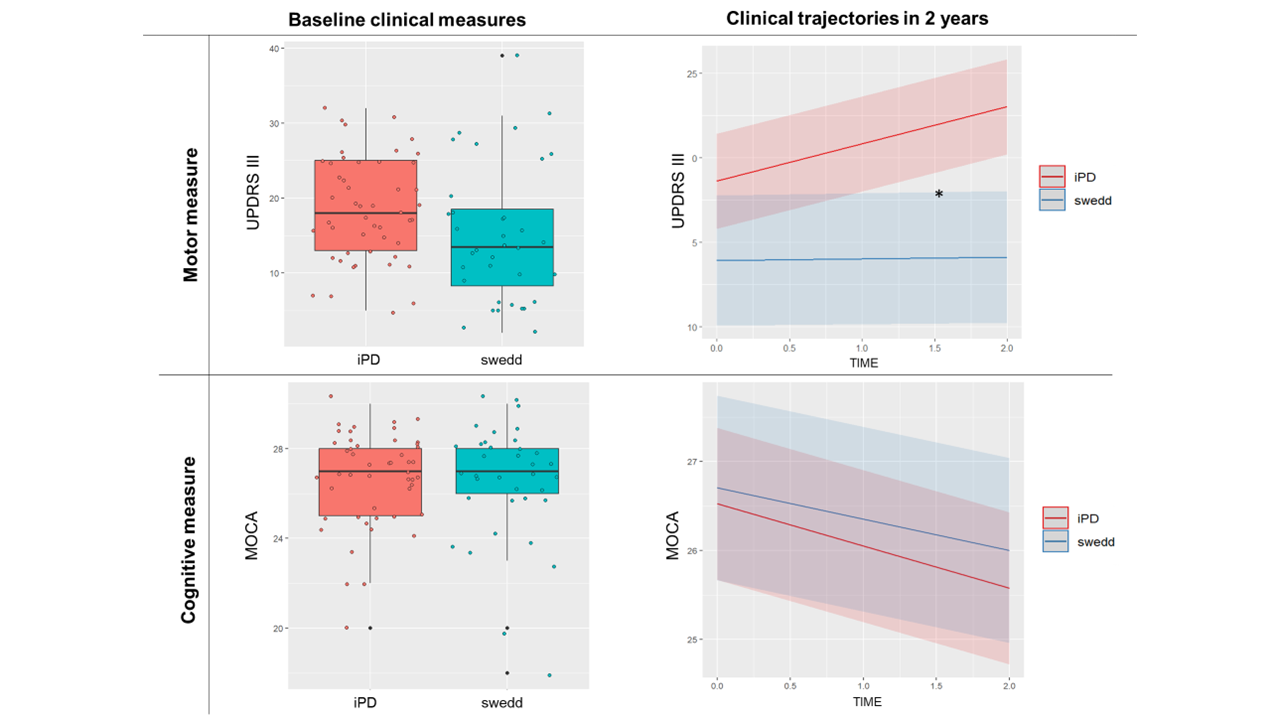


**Figure S2** **Motor and cognitive baseline differences and clinical trajectories over time.** Boxplots in the left column show the motor (UPDRS III) and cognitive (MOCA) scores’ distribution in PD, and SWEDD groups at baseline. SWEDD and PD do not differ from each other in motor and cognitive performance. The right column shows the longitudinal linear mixed models results for UPDRIII and MOCA scores. PD declines faster than SWEDD, that instead remains stable, in the motor symptoms. No differences are found in the cognitive trajectories.


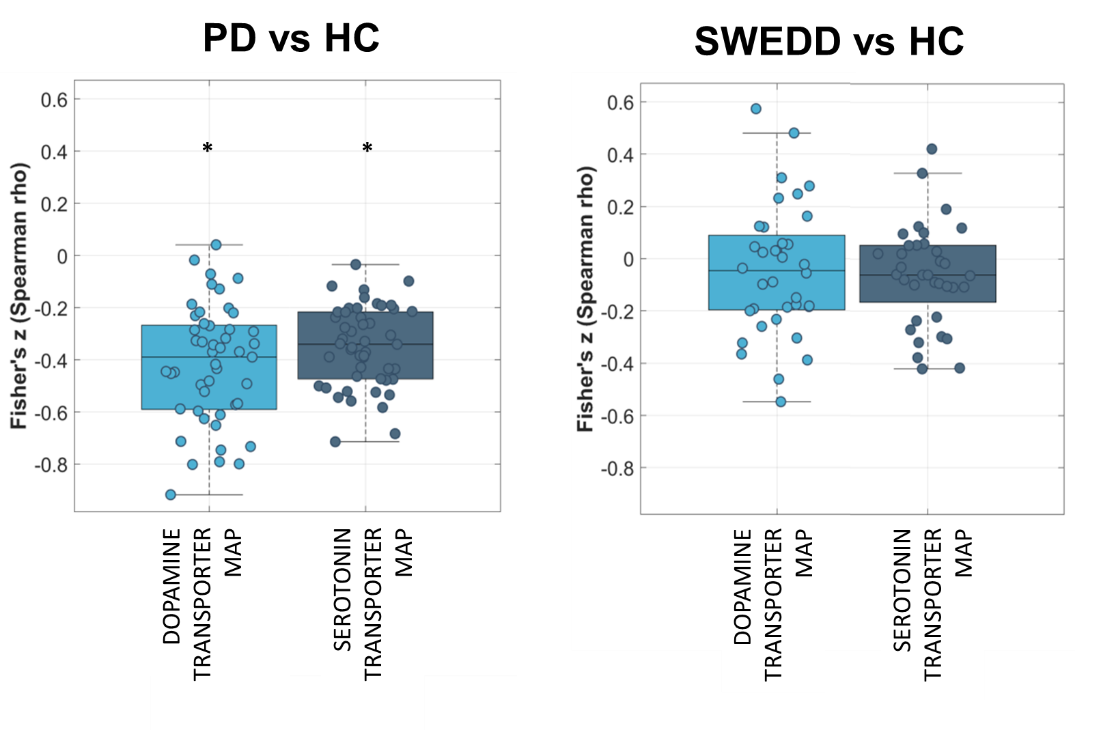


**Figure S3 Results of spatial correlation analysis with neurotransmitter maps for PD and SWEDD**. Fisher’s z-transformed correlation coefficients with respective neurotransmitter maps are displayed for each subject and contrast. ^123^I-FP-CIT binding alterations in PD but not in SWEDD as compared to controls are significantly associated with the topographies of DAT and SERT. Error bars represent the parametric 95% confidence interval of the mean. Exact permutation-based p values (1000 permutations) were computed for all analyses: * indicates p values < 0.001. See text for details. Abbreviations: PD, Parkinson’s disease; SWEDD, scans without evidence of dopaminergic deficits; HC, healthy controls.
